# A Survey of Vehicle to Everything (V2X) Testing

**DOI:** 10.3390/s19020334

**Published:** 2019-01-15

**Authors:** Jian Wang, Yameng Shao, Yuming Ge, Rundong Yu

**Affiliations:** 1 College of Computer Science and Technology, Jilin University, Changchun 130012, China; shaoyameng@hotmail.com; 2 Key Laboratory of Symbolic Computation and Knowledge Engineering of Ministry of Education, Jilin University, Changchun 130012, China; 3 Technology and Standards Research Institute, China Academy of Information and Communications Technology, Bejing 100191, China; geyuming@caict.ac.cn (Y.G.); yurundong@caict.ac.cn (R.Y.)

**Keywords:** V2X, V2X testing, applications, requirements

## Abstract

Vehicle to everything (V2X) is a new generation of information and communication technologies that connect vehicles to everything. It not only creates a more comfortable and safer transportation environment, but also has much significance for improving traffic efficiency, and reducing pollution and accident rates. At present, the technology is still in the exploratory stage, and the problems of traffic safety and information security brought about by V2X applications have not yet been fully evaluated. Prior to marketization, we must ensure the reliability and maturity of the technology, which must be rigorously tested and verified. Therefore, testing is an important part of V2X technology. This article focuses on the V2X application requirements and its challenges, the need of testing. Then we also investigate and summarize the testing methods for V2X in the communication process and describe them in detail from the architectural perspective. In addition, we have proposed an end-to-end testing system combining virtual and real environments which can undertake the test task of the full protocol stack.

## 1. Introduction

The vehicle to everything (V2X) concept uses the latest generation of information and communication technology to realize omnidirectional vehicle to vehicle (V2V), vehicle to infrastructure (V2I), vehicle to pedestrian (V2P), and vehicle to network/cloud(V2N/V2C) network connections [1]. This technology links the various elements of transportation, such as pedestrians, vehicles, roads, and cloud environments. V2X not only can support vehicles to help them obtain more information and promote the innovation and application of automated driving technology, but also can contribute to building an intelligent transport system and promote the development of new modes and new forms of automobiles and transportation services [2]. It is of great significance for improving traffic efficiency, reducing pollution [3], saving resources, reducing the incidence of accidents, and improving traffic management [4]. 

**Figure 1:**
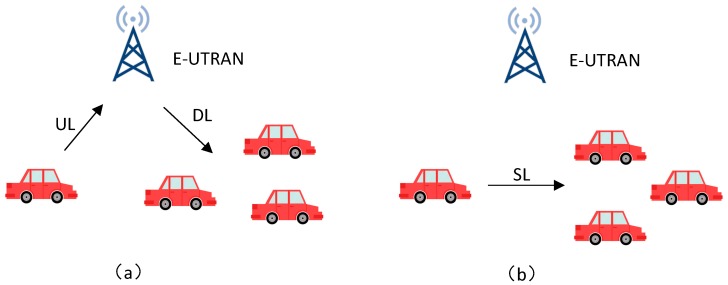
LTE-V2X Communication Modes. (**a**) Uu mode for LTE-V2X (**b**) PC5 mode for LTE-V2X.

Currently, there are two main types of communication technologies used for V2X: Dedicated Short Range Communication (DSRC) and Long Term Evolution for V2X (LTE-V2X). The DSRC system consists of a series of IEEE and SAE standards [5]. At the physical layer and the medium access control (MAC) layer, DSRC uses the 802.11p protocol [6], which simplifies authentication, associated processes, and data transmission before sending data, enabling vehicles to broadcast relevant security information directly to neighboring vehicles and pedestrians. The network architecture and security protocols are defined in IEEE 1609 WAVE [7,8,9]. At the application layer, SAE J2735 [10] defines the message format used for communication, and the J2945/x family of standards defines various scenarios of V2X communication and its performance requirements. LTE-V2X is a wireless communication technology for V2X with high data rate and controlled QoS [11,12], which is based on the evolution of LTE mobile communication technology defined by 3GPP, including two kinds of working modes of cellular communication (Uu) and direct communication (PC5) [13,14,15,16]. The Uu mode uses the existing LTE cellular network to implement V2V communication by forwarding (shown in Figure 1a), and the PC5 mode is similar to the DSRC, enabling direct communication between vehicles (shown in Figure 1b) [17,18]. Additionally, the PC5 interface has been enhanced in many aspects to accommodate exchanges of rapidly changing dynamic information (position, speed, driving direction, etc.) and future advanced V2X services (automatic driving, vehicle platooning, sensor sharing, etc.) [19].

The application of V2X involves many aspects, such as intelligent transportation, intelligent connected vehicles, and automated driving [20]. Different applications have different requirements for latency, reliability, throughput, user density, and safety of the V2X environment [21]. Safety applications and automated driving require extremely low latency and a secure network environment [22,23]. For example, vehicles usually spend most of their time moving at high speed and malicious attackers could cause serious traffic accidents by broadcasting false messages. Malicious attackers may also obtain a vehicle owner’s identity information, vehicle location information, driving trajectory, and so on by interception of data packets [24]. This violates user privacy. The V2X data includes information about roads and geography, which relates to national security. Therefore, security is the top priority for V2X [25]. 

Testing is an important mean to ensure the safety and security of a vehicle. The V2X test is conducted to identify specification flaws, design flaws, and implementation defects over the entire life cycle, and to determine the root cause of the problem [26]. Modeling, analyzing, testing, and evaluating the security threats to V2X can help to improve the security protection capabilities of vehicles and promote the construction of vehicle security systems. In addition, by carrying out testing for V2X, we can pave the way for the commercialization of automatic driving and other applications. 

The structure of this paper is as follows: Section 2 summarizes the types of V2X applications. Section 3 analyzes latency/reliability challenges and security challenges and summarizes the possible security threats. Section 4 describes why we need to do V2X tests and which types of tests should we do. Section 5 lists and briefly analyzes the test objectives and test methods of V2X. Section 6 proposes an end-to-end testing system combining virtual and real environments which can undertake the test task of the full protocol stack.

## 2. V2X Applications

The V2X represents a new cross-industry event involving automobiles, transportation, communications, and the Internet. Various industries and cross-industry alliances have researched the requirements for business applications of V2X. Currently, applications that have been developed or that will be applied in the short term can be classified into three categories: safety applications, efficiency applications, and information services applications [27]. Safety applications refer to applications involving personal safety, such as collision warnings, road hazard warnings, and speeding warnings. Efficiency applications refer to applications that guide owners to drive and improve traffic efficiency, such as green wave speed guidance and congestion warnings. The information services applications refer to applications that provide owners with vehicle-related information to improve the driving experience, such as eCall, traffic information and route recommendations, and automatic parking. With the development of communication technology, the V2X will gradually meet the requirements for advanced automatic driving and applications in intelligent traffic systems. 3GPP defines four types of applications for these advanced application scenarios: Vehicle Platooning, Advanced Driving, Extended Sensors, and Remote Driving [22]. The above applications are all new applications resulting from the development of V2X. However, many traditional mobile applications will gradually enter the V2X industry, such as entertainment services. 

**Figure 2:**
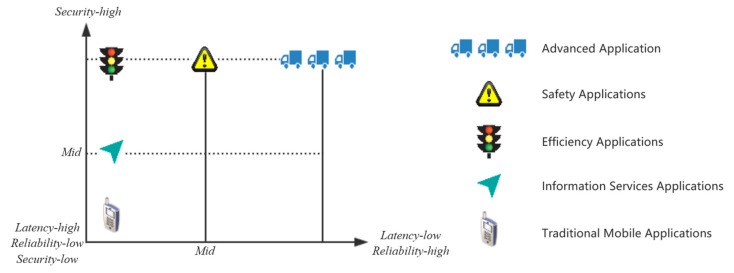
Vehicular Network Applications.

3GPP has developed corresponding requirements for different V2X applications, among which latency/ reliability requirements and safety requirements are the top priorities [23]. The above applications are classified according to latency/reliability and security in Figure 2. The abscissa indicates the application’s requirement for latency and reliability. The larger the abscissa is, the lower the latency required for the application, and the higher the reliability. The ordinate indicates the application’s requirement for security, and the greater the ordinate is, the higher the security level of application security.

## 3. V2X Network Challenges

### 3.1. Latency/Reliability Challenges

The performance of the network is most important for applications which require low latency and high reliability. DSRC uses CSMA/CA to achieve collision avoidance and the ability for multi-user access. With fewer vehicles, DSRC has lower latency and higher reliability, but its performance is opposite in a dense vehicle environment. The latency of LTE-V2X is relatively stable, and the communication delay based on the PC5 interface, which can provide predictable delay and less interference is lower than 100 ms [28]. In the future, 5G networks will provide a communication delay of less than 1 ms while providing a stability of 99.999%, so they will be able support automatic-driving-oriented V2X services.

The V2X network faces many kinds of attacks which could lead to reduced performance. Denial of service (DoS) or distributed DoS (DDoS) refer to intensive use denial attacks by internal or external attackers on target nodes, resulting in the exhaustion of network resources and service resources [29]. They can cause serious problems, such as high latency of communication networks, network unavailability, and unavailability of node services. Jamming attacks and greedy behavior attacks, are examples of DoS attacks [30]. A jamming attack is an attack on the physical layer. The attacker jams the wireless channel through electromagnetic interference, which increases the latency of the V2X communication and reduces the network’s reliability [31]. A greedy behavior attack refers to a network node that violates the rules of channel access and occupies too many channel resources, thereby reducing the performance of other nodes and causing network congestion [32].

### 3.2. V2X Security Threats

The network security is an important part of V2X technology and the threats faced by V2X are divided into four aspects: mobile terminal security threats, V2X service platform security threats, V2X communication security threats, vehicle network data and privacy threats [33].

The combination of mobile smart terminals such as mobile phones and V2X can not only provide information and entertainment services for car owners, but also provides the function of remotely controlling vehicles. Mobile smart terminals typically connect to wireless networks such as in-vehicle Wi-Fi networks or Bluetooth, which provides malicious attackers with a springboard to the in-vehicle network. Moreover, applications on the mobile terminal are vulnerable to hackers because of its low threshold of development and easy accessibility.

The cloud service platform not only faces the problems of traditional network cloud platforms, but also has a weak identity authentication problem caused by the principle of mutual trust in V2X communications [33]. Whether the data in the cloud will be leaked is a major problem [34]. Moreover, the V2X cloud platform contains data about vehicles, roads, and pedestrians. If these data are leaked, they could cause significant losses. Owing to the high-speed mobility of vehicles, identity authentication and establishing a trusted connection with the cloud is a difficult problem. How to identify false data uploaded by an attacker and how to uniformly manage different types of data uploaded by different vehicles are also challenges faced by the cloud platform [35].

Because of its wireless transmission properties, the V2X network is particularly vulnerable to attacks. Therefore, communication security is very important. The security attributes include authentication, availability, data integrity, confidentiality, non-repudiation, real-time constraints, and attacks against these security attributes are as follows [30,36,37,38,39]:

- Authentication: Sybil attack, GPS spoofing/position faking attack, Node impersonation attack, etc.
- Availability: DoS attack, DDoS attack, Jamming attack, black hole attack, etc.
- Data Integrity: Masquerading attack, Replay attack, etc.
- Confidentiality: Eavesdropping attack, Traffic analysis attack, etc.
- Non-repudiation: Loss of events traceability, etc.
- Real-time constraints: Timing attack, etc.

According to the attacker’s network location, attackers can be divided into insiders and outsiders. Insiders can communicate directly with other vehicles, but outsiders cannot. According to the purpose of attackers, they can be divided into malicious attackers and rational attackers. Malicious attackers destroy the network, not considering personal interests, while rational attackers do so to achieve personal benefits. According to the attack mode, attackers can be divided into active attackers and passive attackers. Active attackers actively send packets, but passive attackers only monitor a network. According to the scope of activities, attackers can be divided into local attackers and extended attackers. Local attackers only act within a limited range of activities, while extended attackers expand their range of activities by controlling other nodes [36,40].

Compared with the traditional network, the data on the V2X network is more open, so it is easier to expose more privacy data. Attackers can passively intercept user data or actively invade vehicles or cloud service platforms to steal information. In addition, mobile terminals such as smart phones also have the risk of privacy exposure. User privacy data such as the owner’s name, plate number, vehicle speed, and driving route should be prevented from being acquired by others. However, some user privacy data must be open to trusted third parties such as police and accident rescue to ensure timely handling of emergencies such as accidents while being able to detect and track malicious attackers [41]. At present, V2X is in the initial stage of development. The data management and privacy protection systems are still in the process of being perfected. It is necessary to discuss and refine key issues such as which data can be collected, how data is used, and whether it can be shared with third parties [33].

## 4. The Need for V2X Testing

Since vehicles usually spend most of their time moving at high speed, it may have serious consequences when an accident happens and even threaten the safety of the driver and passengers [42]. Safety always has the highest priority, so how to ensure vehicle safety has always been a serious topic. In the field of traditional automobiles, various testing and evaluation systems have been established in all world countries. Testing is an indispensable part of the Internet of Vehicles which is a new thing for us. If a vehicle receives erroneous data in a specific environment such as a highway or a crowded area, it may cause false triggering of a safety application, resulting in a serious traffic accident. Testing can ensure the reliability of the communication, thus ensuring the safety of the entire V2X environment. Because of the high requirements for security of the V2X, the priority of V2X testing is also high.

There are many problems in the Internet of Vehicles, which seriously hinder the development of vehicle networking technology and commercialization. Firstly, in special scenarios such as intersections or traffic jams, the density of vehicles is very large. The sheer number of users puts tremendous pressure on wireless communications, so it easily causes communication congestion [43]. Secondly, the vehicle has the characteristics of high mobility and rapidly changing network topology [44], which brings great difficulties to data transmission, routing, etc [45]. For example, two vehicles traveling in opposite directions will drive out of communication range within a few seconds. These communications have the requirement of low latency and high reliability for the Internet of Vehicles [46]. Finally, how to design and develop a good application is also an important issue for the Internet of Vehicles [47]. Before developing an application, developers need to spend a lot of time trying to determine the application scenarios. In addition, how to ensure the safety and effectiveness of an application is also an important issue [48].

Internet of Vehicles is a new cross-industry thing involving many industries such as automotive, communications, transportation, etc. As the name implies, V2X needs to connect all vehicles together, so the interconnection and interoperability are important attributes [49]. In the Internet of Vehicles, if a vehicle cannot understand the data sent by another vehicle with different brand, it will cause the lack of the information, and greatly reduce the meaning of V2X. Besides it may also lead to serious accidents resulting in unnecessary loss. At present, countries in which the V2X is growing up have been developing communication standards to help vehicles and other transportation participants to communicate unimpeded. These standards can also achieve understanding between different brands of vehicles and different intelligent transportation infrastructures, to ensure interconnection and interoperability of the V2X.

Testing aims to ensure safe and effective use of the Internet of Vehicles. Different testing methods are adopted for different needs. Communication standards, as a common language among vehicles, infrastructures, clouds, etc., can help vehicles communicate with other traffic participants accessibly, enabling the interconnection and interoperability. Interoperability testing can ensure information exchange and coordination between devices [50]. The protocol conformance testing aims to verify the conformity of each manufacturer’s terminals with standards [51]. They lay a foundation for the interconnection and interoperability between different manufacturers’ devices. Different V2X applications have different communication performance requirements [52], such as automatic driving needing extremely low latency, and video entertainment applications requiring larger bandwidth. The performance testing is mainly used to test the performance of the V2X network in different scenarios, including latency, communication range, packet loss rate, etc., to ensure that communication can meet the needs of the applications. Ensuring the safety, effectiveness and reliability of V2X applications is also an important goal of testing. The function testing can determine whether an application is valid, whether it can be triggered correctly in a specific scenario, and whether it can ensure vehicles safety. Malicious applications will be removed after function testing. In general, we need to test functionality, performance, interoperability and consistency of the V2X terminal.

## 5. V2X Testing Methods

At present, V2X technology is in the exploratory stage. The traffic safety problems and information security problems brought about by its application have not yet been verified, so testing is an important part of V2X. Function testing, performance testing, and communication protocol conformance testing are mainly used to meet the testing requirements for latency and reliability. Security protocol consistency, gateway testing, penetration testing, and accelerated testing can find vulnerabilities and potential risks and its applications to ensure its security. After laboratory testing, V2X applications must undergo field testing before they can be used commercially. Field testing is mainly used to evaluate the performance of V2X applications in a real environment and to meet the performance and function requirements in a large-scale environment.

### 5.1. Conformance Testing

Protocol conformance is the basis of V2X communication. Only by meeting the protocol conformance can we ensure the interoperability between vehicles, pedestrians, RSU, cloud platform, and other participants, which is the basis for developing various types of V2X applications. The protocols can be divided into two major categories. One is the communication protocol, which stipulates the data format and interaction flow of the communication processes in a V2X network, and the other is the security protocol, which stipulates the security processes such as certificates and authentication. Communication protocol conformance testing can ensure the interconnection and interoperability of devices [53], and security protocol conformance testing ensures security. Therefore, protocol conformance testing is a must-have requirement for all telematics devices.

**Figure 3:**
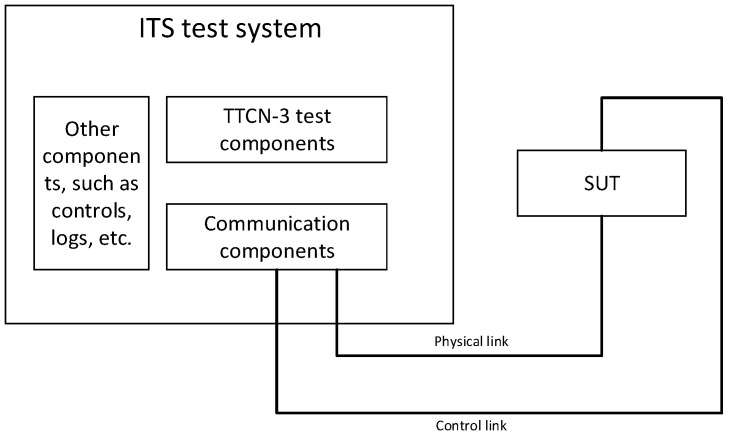
Abstract Test System [54].

ETSI stipulates an abstract test system for V2X conformance testing [54]. There are many ways to implement this abstract test system. ETSI recommends using TTCN-3 [55] to implement the abstract test system, as shown in Figure 3. This implementation conforms to ISO 9646 [56].

**Figure 4:**
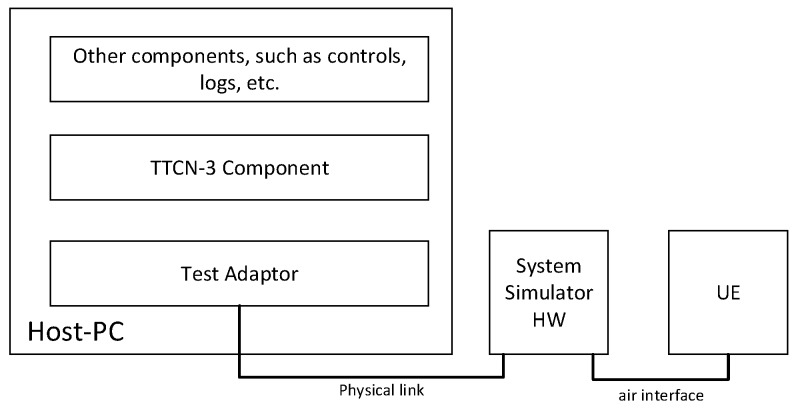
Test System Architecture Based on TTCN-3 [57].

3GPP provides a TTCN-3 based test system architecture [57], as shown in Figure 4, which is similar to the ETSI abstract test system. The difference is that the TTCN-3 test system (Host-PC in Figure 4) communicates with the device under test (UE in Figure 4) through the System Simulator hardware (HW). The test system is combined with the System Simulator HW, which is equivalent to a complete and controllable communication device that can fully test a device under test according to test cases by automatically or manually simulating communication processes.

In addition, ISO TS 20026 [58] also describes several similar ITS test architectures, which are generally indistinguishable from the above test architecture.

In order to support the conformance testing and enhance the completeness, test specifications have been proposed. For example, the Certification Operating Council (COC) under the U.S. Department of Transportation has established a series of specifications for conformance testing. In China, the C-V2X WG under IMT-2020(5G) promotion group also have established specifications for conformance testing, function testing and etc. Fouchal [59,60] designed a set of tools that could be used in order to check the conformance of a Cooperative-Intelligent Transport System (C-ITS). But there are still many challenges during the process of conformance testing. Firstly, due to the complexity of the standards, conformance testing needs to test every field in that standards which mean we need to design a large number of test cases. If a tester manually does a test, a lot of time will be wasted and there may be many mistakes during the testing process. So, the automated testing system has been developed. Its advantages are saving time and decreasing mistakes. But additional development needs to be done by the equipment developer because the automated testing system need control the device under test through a test control interface (TCI). The more complicated the test process is, the more additional development may be. So how to push the manufacturers to do the automated testing is a big question. Secondly, how many test cases are sufficient is another question. Test cases in conformance testing not only need to cover all the fields of the standards, but also need to test the understanding of the specific fields. For example, the representation and unit of longitude and latitude maybe differ. These confusions are mostly caused by the vague description in the standards and the conformance testing is a nice feedback to these standards. Finally, the above implements for conformance testing are all black-box, and they lack information or knowledge of system under test (SUT) [61]. Therefore we can’t confirm whether a device under test transmits data by its protocol stacks. If the test cases have vulnerabilities, the manufacturers may develop a special program only for conformance testing instead of the part which is difficulty to develop in their device.

### 5.2. Function Testing

Application function testing can be used to determine whether an application can be triggered and take reasonable actions in different scenarios. It can guarantee the reliability and effectiveness of V2X applications. According to the environment, function testing can be divided into two types: laboratory testing and field testing. There is no doubt that the field testing is closest to reality. But if we want to do the V2X field testing, the testing scenarios will cost a lot of time and money. Besides, most of the scenarios are hard to build and the number of scenarios is huge. So a virtual function testing system in the lab is a perfect solution.

A virtual function testing system can be built only by software simulators, which means that all the testing scenarios are virtual. Aramrattana and Larsson [62] presented a simulation framework for testing and evaluation, which was aimed to test platooning. They used VTI for driving simulation and Plexe for traffic and network simulations. All the simulations were virtual and the framework had advantage that it was cheap to build and easy to extend its function such safety warning. But it depended severely on the models such as communication model and traffic model. If a model didn’t accurately reflect the real situation, the result of testing could be wrong leading invalid testing. Mittal and Savita [63] contrasted several simulators for VANETs which were old but some of them has been updated. They did the comparative study from the aspect of software characteristics, graphical user interface, accuracy of simulation, ease of use, popularity, input requirements and output visualization capabilities. Accuracy of simulation and ease of use were the most important for a simulator because they impacted directly on the testing results. Kim and Kim [64] had proposed a V2XREF of V2X Runtime Emulation Framework that can be used to perform the evaluation and validation of vehicle safety applications and safety systems along the various safety scenarios. Their emulation framework was also based software simulation and could be used in function testing. Schiller and Alois [65] had presented a new virtualization-based approach for emulating VANET by which application function testing could be performed. Choudhury [66] designed the simulation environment for V2X protocol and application testing. The test environment is divided into three parts, traffic simulators using VISSIM, network simulators using NS-3, and application simulators using MATLAB. The test environment adopts software simulation, so the underlying physical communication process cannot be tested. And because the tested application is not real and complete application software, it can only be tested against the application core algorithm and performance requirements. Ahmed [67] designed a test environment for testing V2X applications including three subsystems: input subsystem using NS-2, core subsystem to manage data and client framework subsystem providing a virtual test environment. Ming and Zhao [68] built a general testing framework based on Veins for securing VANET applications. Ribeiro and Gonçalves [69] tested a Platooning Management Protocol with VSimRTI framework.

The virtual simulation has its limitations which can’t accurately reflect objective facts. Traditional Hardware-in-the-Loop (HIL) can be used in the V2X testing. Buse [70,71] proposed the Ego-Vehicle Interface (EVI) to integrate these very different types of simulators including HIL simulators and VANET simulators. Szendrei and Varga [72] designed a hardware-in-the-loop (HiL) V2X simulation framework to offer a cost-efficient and simple toolset. In the function testing with HIL, the device under test could be seen as a black-box and the testing environments were generated by the simulators. Its advantage is that we can test the V2X devices instead of the applications or algorithms. But it also faced the above problems because the testing environments were still virtual.

**Figure 5:**
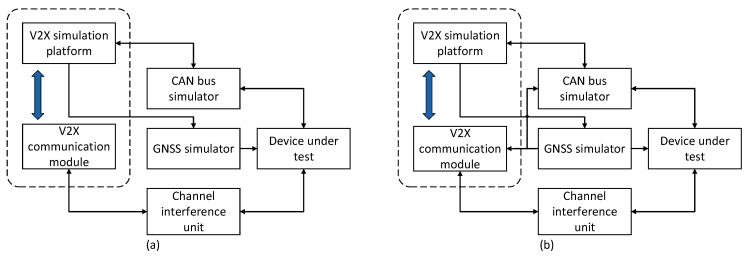
Function Test System Architecture. (**a**) the V2X simulation platform directly generates application messages and sends them to the V2X communication module; (**b**) the V2X communication module generates corresponding application messages according to the simulation data from the GNSS simulator and the CAN bus simulator.

Our team is building a function testing system which extends the HIL methods. The system uses communication devices instead of the network simulator. The architecture is shown in Figure 5, including the V2X simulation platform, V2X communication module, GNSS simulator, channel interference unit, and CAN bus simulator. The V2X simulation platform performs application scenario simulation, test cases management, test result analysis, etc. It is used to generate dynamic simulation data of the vehicle, such as vehicle speed, position, distance between vehicles, obstacles, traffic scenes, etc., and manages the entire test activity. The V2X simulation platform generates a virtual traffic scenario based on the test cases. The scenario includes a host vehicle (HV) and at least one remote vehicle (RV). The host vehicle refers to the vehicle under test in this scenario, and the RV is used to assist the HV to trigger applications. The V2X simulation platform is connected to the V2X communication module through an Ethernet interface or similar to control the transceiver behavior of the V2X communication module. It also interconnects with the GNSS simulator and the CAN bus simulator through a serial or similar port, and sends the simulation data to the V2X communication module and the device under test. The V2X communication module is used to simulate a RV in a virtual traffic scenario to generate application messages such as a basic safety message (BSM), and then sends it to a device under test through the channel interference unit. Meanwhile, the V2X communication module can also receive application messages sent by the device under test. We have two methods to obtain the application messages of the V2X communication module. One involves the V2X simulation platform directly generating application messages and sending them to the V2X communication module, as shown in Figure 5a. The second involves the V2X communication module generating corresponding application messages according to the simulation data from the GNSS simulator and the CAN bus simulator, as shown in Figure 5b. The channel interference unit simulates the communication environment, such as signal attenuation, interference, and so on. The device under test is used to simulate the HV in a virtual traffic scenario and runs V2X applications relying on simulation data. If the application is triggered, the device under test will generate a control action or warning information, which will be fed back to the V2X simulation platform through the CAN bus simulator. At this time, the HV will perform actions such as braking and issuing a warning, in the virtual traffic scenario.

There may be many problems in the function testing. Firstly, time synchronization is a big challenge for testing system. Because vehicles usually spend most of their time moving at high speed and the frequency of message transmission is very high, the unsynchronized vehicles will lead to trigger the applications wrongly. How to integrate real-time simulators and non-real-time ones guaranteeing time synchronization should also be considered before building the function testing system. Secondly, extra communication simulation delay that is the simulating delay minus the real delay such as V2V delay should be low. The extra delay may be generated due to the communication models. If the extra delay is high, the communication simulation can’t reflect the reality. Finally, testing scenarios which is the key of function testing should cover special situations such as traffic jams, highway, mountain area and etc. In these extreme scenarios, application may perform bad.

### 5.3. Performance Testing

Performance testing is an important mean to guarantee the latency and reliability requirements of V2X applications. It mainly includes end-to-end communication delay testing, packet delivery success rate testing, and parameter testing, such as signal strength under different channels. Through performance testing, testers can understand the effects of basic network communications, and further determine whether the performance of network communications can support V2X applications.

Qin and Meng [73] implemented the 802.11p and 1609.3 protocols and tested the latency and packets loss in real filed. Carpenter and Sichitiu [74] developed a simulation to test the routing throughput, end-to-end delay and packet delivery ratio of a protocol in several VANET scenarios. Hiromori and Umedu [75] proposed a method for efficiently carrying out protocol testing for a set of designated node density distributions and their variations for VANET applications, network throughput, packet loss rates and etc. Vongpasith and Wang [76] analyzes routing protocol on the aspect of packet delivery ratio, average end-toend delay and throughput using a NS-2 simulator. Bouchra and Hicham [77] evaluated the packet delivery ratio, average end-to-end delay and bandwidth using a simulator based on NS2 and MOVE. Utkarsh and Raghavendra [78] testing the performance of the IEEE 802.11p standard for varying node density and data transfer rate by means of simulations using NS-3. Huang and Zhao [79] studied the performance of DSRC based on Safety Pilot Model Deployment Data. Shi and Lu [80] evaluated the communication performance of Intersection Collision Warning(ICW) in field. Kawasaki and Onishi [81] investigated the performance of PC5-based and Uu- They are not the authors’ surnames, please confirm.based LTE for crash warning application.

**Figure 6:**
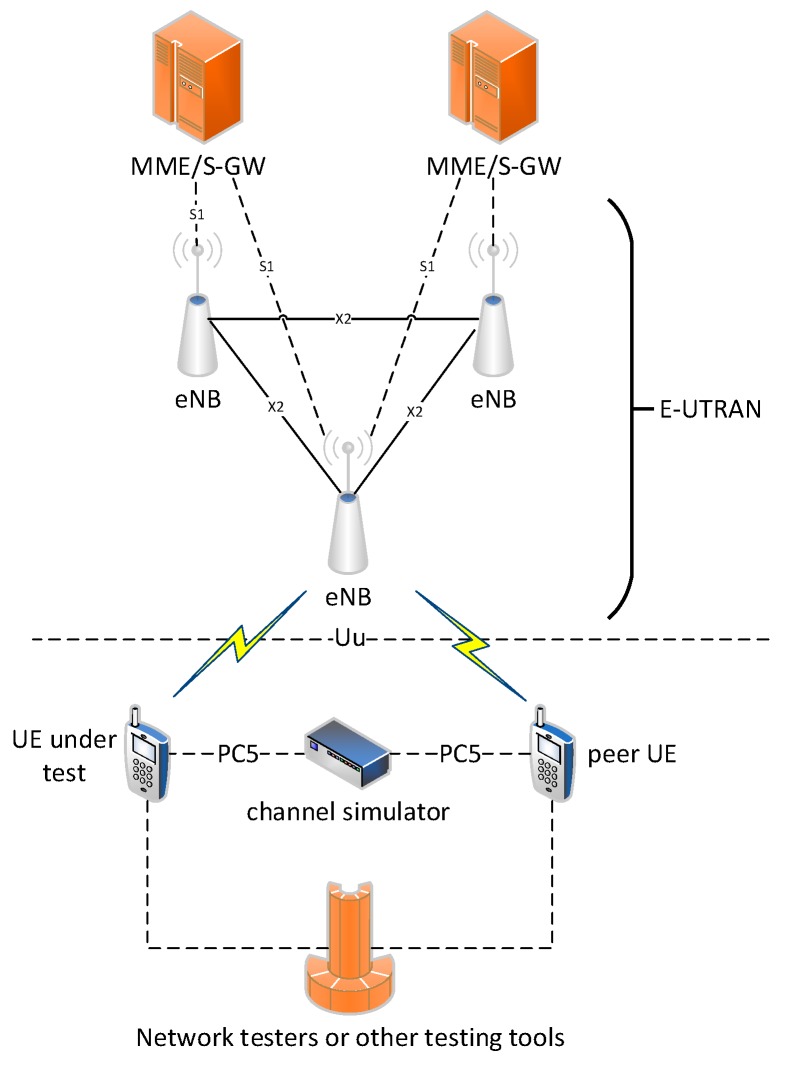
Terminal Performance Test System Architecture.

Now we have built a performance test-bed for black-box testing. The architecture of the test-bed is shown in Figure 6. The test system consists of three parts, including UEs (also referred to as user equipment, including the UE under test and the peer UE for simulating the process of transmission), the evolved core network (i.e., the MME/S-GW in Figure 6), and the evolved UMTS terrestrial radio access network (E-UTRAN). The channel simulator is used to simulate wireless channel propagation over the PC5 communication link. Network testers or other testing tools (such as test software developed by the device provider) are responsible for managing the testing process and receiving testing data. The UEs can connect with the E-UTRAN system through the TD-LTE air interface (i.e., the Uu interface). The system can test sidelink basic transmission under Mode4 and Mode3, and IOT test of RRC protocol performance. Based on the timestamps and packet numbers in the packet header, the transmission delays and packet delivery success rates of the packets are measured.

Most of the above works are mainly focused on the performance of a protocol using a simulator. However, equipment from different manufacturers may perform differently. So we need to test them using special testing methods and tools in the lab or filed. The lab testing could simulate a wide variety of performance testing environments, but it relies heavily on the model in simulators. The field testing is more realistic, but the extreme case is quite rare. Therefore, the performance testing meets the question of how to balance the lab testing with the field testing. The performance of V2X communication is also an important issue in the case of obstacles (such as buildings). To test performance in this scenario, modeling analysis and software simulation can be used [82,83,84,85]. In addition, lab testing can use channel simulators to generate non-line-of-sight communications. Testing performance in the case of obstacles does not require complex test scenarios, and only two communication terminals are required. Therefore, field testing is superior to lab testing.

### 5.4. Vehicle Gateway Testing

**Figure 7:**
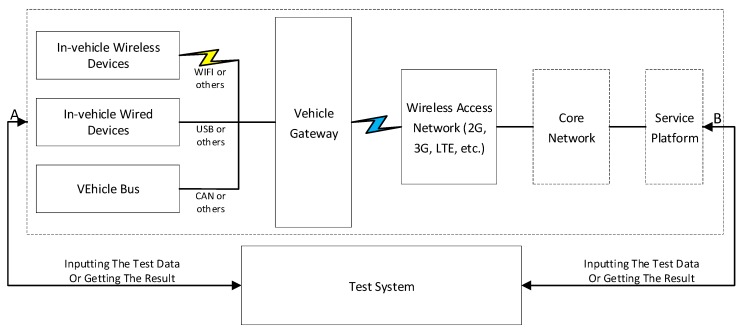
Vehicle Gateway Testing Architecture.

Vehicle gateway testing is a mean to ensure that the vehicle gateway is running correctly, so it can meet the needs of V2X network security. The test architecture of the vehicle gateway is shown in Figure 7. The test architecture consists of two parts: the system under test and the test system. The system under test is composed of vehicle gateways and other systems or devices (including in-vehicle devices, networks, and service platforms), as shown by the dashed boxes in Figure 7. The test system is connected with the A-side and B-side of the system under test in Figure 7. According to test cases, the test system inputs the test data to the system under test, which generates the corresponding response according to the input of the test system. The test system conducts tests by analyzing the differences of the expected results and the actual results obtained from the system under test. The test system supports inputting the test data and obtaining the results from the A-side and B-side in Figure 7 simultaneously.

### 5.5. Penetration Testing

Penetration testing is a method of simulating a malicious attacker’s methods and testing the security of the target system. It is a critical step in the development of the V2X. The penetration testing needs specific tools by which vulnerabilities of the V2X can be scanned [86]. The effectiveness of penetration testing relies on the skills and experience of testers [87], and it involves many disciplines, such as software, electronics, radio frequency (RF), and cryptography [26]. Therefore, penetration testing can be performed by personnel on an independent internal Cybersecurity test team or by outside third party engagement [88]. The penetration testing can be divided into three categories: white box testing, black box testing, and gray box testing [26,89]. In black box testing, the tests have no information regarding the tested system in advance, and they need to look for vulnerabilities from the perspective of the attacker. Its advantage is that the testing process is more realistic [90], but the disadvantage is also obvious—more testing time is needed. In white-box testing, testers can obtain information such as the design specifications of the system under test and code implementation in advance, making it easier to identify problems. However, the V2X is a complex system, and many design details should not be provided to testers, thus greatly reduces the advantages of white-box testing. Gray box testing is a mix of white box testing and black box testing which can obtain some information of the system under test and mainly adopts the white box test method. For the parts which testers cannot obtain information, the methods for black box testing are used. There are three types of specialized penetration testing: interface testing, transportation testing, and system testing [91]. Interface testing targets interfaces among the vehicles, mobile terminals, roadside units. Transportation testing focuses on misuse issues and design flaws in communication protocols and weak cryptographic schemes. System testing examines the implementation flaws, insecure system settings and other known vulnerabilities of the vehicle gateways, vehicle systems, cloud systems, mobile terminal OSs, etc. And special penetration testing tools and techniques are needed to cover the above testing requirements.

The penetration testing process includes creating a threat model, designing a test plan, executing test cases, and generating test reports [92]. Creating a threat model can be based on the V2X security threats described in Section 3.2. Designing a test plan must guarantee availability of personnel or devices, meeting timelines and deliveries, comprehensive test cases, and adequate test tools. The process of executing test cases mainly focuses on system dependencies, user interfaces, system design, and system implementation. The test report is generated from the perspective of vulnerability reappearance, vulnerability severity assessment, and scenarios where malicious attackers exploit vulnerabilities.

Penetration testing generally adopts an iterative process. When a system vulnerability is discovered through testing, it is likely to expose additional problems. Therefore, testers can perform iterative testing based on the discovery of vulnerabilities. In addition, penetration testing can be combined with several other automated testing tools, using static analysis and dynamic analysis methods, reducing time and costs [93,94]. The test frameworks discussed in Section 5.1, Section 5.2 and Section 5.3 can also be used for penetration testing, for example, using test simulation devices for Sybil attacks, Replay attacks, etc., using channel jammers to simulate channel congestion scenarios. In addition to the test frameworks, those simulators mentioned above can be seen as the system under test. Then, testers can perform penetration testing to the simulators. 360, Visual Threat, Rapid7 and other companies have formed security teams to provide penetration testing services for communication protocols, mobile terminal APP, Cloud API, firewalls and etc.

### 5.6. Accelerated Testing

To solve the slow testing process of a vehicle, the accelerated test method can effectively reduce the cost and time spent on the vehicle reliability verification process [95]. Accelerated testing requires a significant amount of real-world data, and critical scenarios are used to analyze potential vehicle problems [96]. The analysis results will be applied to additional scenarios and the above process will be repeated. Some technologies such as importance sampling are used to accelerate the testing process [97]. Therefore, accelerated testing can greatly speed up a vehicle’s test process. Accelerated testing can be used in lab simulations, human-in-the-loop tests with driving simulators, hardware-in-the-loop tests, or field testing [98]. Accelerated testing can also be used to test the reliability of the V2X in working conditions such as heavy rain, fog, and other extreme weather, and the network communications performance during traffic jams.

Some challenges should be considered before taking accelerated testing. One is that it performs badly in the filed because it’s hard to build the real-world environment. The other is how to build the model with the real-world data and how to ensure that iterative data is valid. From the perspective of communication, accelerated testing can be used in function and performance testing in the lab. For example, testers build the application scenarios according to the real world and generate new scenarios with the accelerated method.

### 5.7. Field Testing

The V2X network and its applications must be subjected to field testing and large-scale demonstration running before being officially promoted and used, such as the US’s Safety Pilot Model Deployment [99], Security Credential Management System (SCMS) for vehicle-to-everything (V2X) communications [100], HarborNet [101], M-City, Safe intelligent mobility (sim^TD^) [102], Korea Intelligent Transportation Demonstration in Jeju Island, China’s 5+2 Internet of Vehicle Pilot Zones, etc. Waymo, Google’s subsidiary for automatic driving, has conducted more than four million km of road testing [103]. Tesla has used simulators to test over several million km in conjunction with actual roads. Chinese Internet companies such as Tencent and Baidu have also used simulators and road-testing methods to conduct extensive testing in demonstration zones. Unlike the traditional mobile applications, the V2X applications are deployed in the vehicles. So the tests of them need to be done in the field which is a big cost for the developer. In general, field testing requires a large number of basic network facilities and transportation facilities, test vehicles, testers, etc. So how to effectively reduce the cost of testing is particularly important [104].

**Figure 8:**
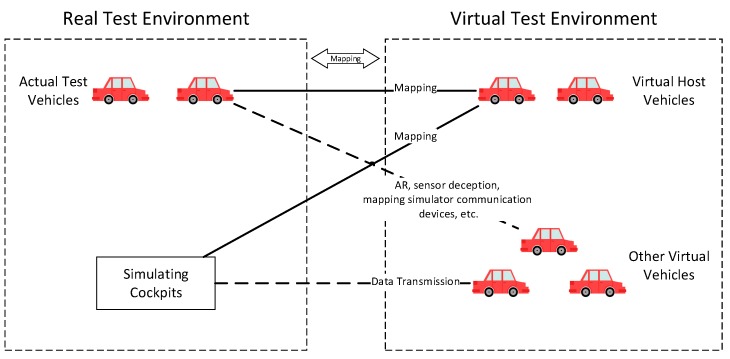
Parallel Testing Architecture.

Parallel testing can effectively reduce the cost of large-scale testing [105]. It belongs to the closed-loop hardware in the loop (HIL) test, which is mainly aimed to test the communication process and applications in the vehicle network and can provide a test environment for automatic driving. As shown in Figure 8, parallel testing can build a virtual test field based on an actual test field or create a complete virtual test field. The virtual test field includes a large number of virtual vehicles and virtual host vehicles. The host vehicles establish mappings with actual test vehicles or simulation cockpits. Other virtual vehicles use augmented reality (AR), sensor deception, mapping simulator communication devices, etc., to communicate with the test vehicles or simulation cockpits. Through the mapping of this virtual test environment to the real test environment, a large-scale test for V2X is realized.

### 5.8. Testing Tools

Testing tools are the basis of V2X testing. Many companies, organizations and research institutes have designed and developed a variety of testing tools. Spirent developed the 802.11p-based V2X Emulator to test V2X functionality and performance, and to support conformance testing of the WAVE protocol stack. OmniAir in the U.S. have developed DSRC conformance testing specifications and have conducted testing and certification work. In Europe, the 5GAA organizes cross-industry companies to establish a C-V2X test and evaluation system and has conducted corresponding large-scale field testing. Neusoft, Jilin University, etc. have developed cooperative perception sensing components and tools for testing in accordance with national standards. The V2X simulation runtime infrastructure (VSimRTI) was designed for the assessment of new solutions for Cooperative Intelligent Transportation Systems [106].

**Figure 9:**
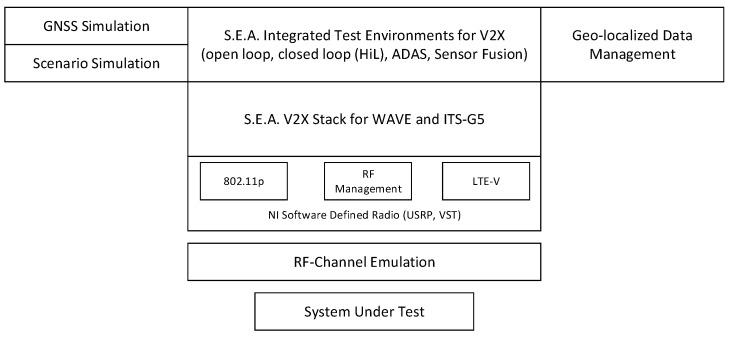
S.E.A. Testing Tools.

Science & Engineering Applications Datentechnik Gmbh (S.E.A.) provides a complete V2X testing tool chain, including a series of V2X communication products and test systems, and provides customizing, support, and consulting services. Figure 9 shows the S.E.A. test tool set. The testing scope includes the application layer, network layer, access layer, and physical layer. The test architecture is similar to that discussed in Section 5.1, Section 5.2 and Section 5.3, but it is divided into two parts: open-loop test and closed-loop test. The open-loop test uses pre-designed scenarios to test the intrinsic behavior of the device under test. The closed-loop test adopts interactive driving scenarios to test platooning, dynamic controlling, and other interactive activities. NI also provides V2X test services, including PXI systems for testing RF, m3 systems for testing GNSS, HIL test systems, and wireless test systems. For the applications and traffic scenarios, simulators are the main technology. Whether a simulator is excellent depends on the accuracy between its simulating output and the real world. For the communication, simulators are used to test the protocol. But in the actual testing process, communication devices usually be black-box and the channel may be simulated by testing tools. As for field testing, the communication environment is hard to build, because it is very expensive to build an extreme scenario such as traffic jam. Test tools mainly collect data in the field testing by which testers analyze the performance and function of a real vehicle application or device.

## 6. End-to-end Testing System Combining Virtual and Real Environments

From Section 5 we can see that current testing methods are independent and each of them have only one or two testing purposes, so we have proposed an end-to-end testing system combining virtual and real environments which can undertake the test task of the full protocol stack. The testing objects can be application function, protocol conformance, communication performance and etc. The system can be divided into three parts: scenarios, communication and applications.

The scenarios are the basis of the system and can be virtual, real or mixed. The virtual scenarios are used in the simulators to build the virtual testing environment. The real scenarios mean that the tests will be done in a field. We can use the real-world data to build the scenarios or map virtual objects to real scenarios which we call the mixed scenarios. One or more testing objects will be put into those scenarios. The communication connects all entities in the scenarios. The communication between virtual entities can be simulated by tools such as NS-3. But the communication between a virtual entity and a real entity is hard to handle. We can map the virtual entity to another real entity to transmit data in the real world or map the real entity into the virtual scenery to communicate with a simulator. Thus the communication has only two types: the virtual or the real. We also divided the applications into two types: virtual or real. The virtual application is the algorithm driven by a simulator in the virtual scenarios which can increase the integrity of the scenarios. The real application is one driven in a real device which is usually used as the background object.

We can combine all types of scenarios, communication or applications to build a test environment. For example, if we want to do a performance test for application layer protocol with low cost, we can choose the virtual communication whose network layer, access layer and physical layer are simulated. And forward collision warning in an on-board unit seen as a black-box can be tested with mixed scenario, real communication and virtual background application shown in Figure 5 which is easy to use and the cost is low.

## 7. Conclusions

As new technology, V2X not only provide a more comfortable and safer traffic environment, but also are important for improving traffic efficiency, reducing pollution, and reducing accident rates. The main V2X communication technologies are DSRC and LTE-V2X. There are many V2X applications, and their application requirements mainly focus on latency/reliability and security. Latency/reliability is threatened by the network performance problems and multiple types of malicious attacks. Security faces mobile terminal security threats, V2X service platform security threats, V2X communication security threats, vehicle network data and privacy threats. Testing is an important part of V2X. First, we describe the abstract test system and then introduce test methods from three perspectives: function, performance, and conformance. We then focus on testing methods such as vehicle gateway testing, penetration testing, and accelerating testing, and analyze the requirements for field testing. Finally, we have proposed an end-to-end testing system combining virtual and real environments which can undertake the test task of the full protocol stack.
